# Dasatinib-Associated Gastrointestinal Bleeding and Colitis: A Narrative Review with Illustrative Cases

**DOI:** 10.3390/jcm15208086

**Published:** 2026-10-18

**Authors:** Nguyen Thi Mai Nhi, Shyn Yi Tan, Justin Wen Hao Leong, Valerie Shu Xian Yeap, Bochao Jiang, Aloysius Jiawei Loo, Jing Yuan Tan, Denise Tan Yan, Yeow Tee Goh, Kok Hing Lim, Yu Bin Tan

**Affiliations:** 1 Department of Gastroenterology and Hepatology, Singapore General Hospital, Singapore 169608, Singapore; 2 Duke-NUS Medical School, Singapore 169857, Singapore; 3 SingHealth Polyclinics—Outram Polyclinic, Singapore 168937, Singapore; 4 Department of Haematology, National Cancer Centre, Singapore 168583, Singapore; 5 Division of Pathology, Singapore General Hospital, Singapore 169608, Singapore; 6 Cancer Genetics Service, Division of Medical Oncology, National Cancer Centre, Singapore 168583, Singapore

**Keywords:** dasatinib, gastrointestinal bleeding, colitis, tyrosine kinase inhibitor, drug-induced colitis, post-polypectomy bleeding

## Abstract

**Key Points:**

**What unusual bleeding event did we observe in a dasatinib-treated patient?**

- We describe clinically significant delayed bleeding after cold snare polypectomy of a diminutive (≤5 mm) colorectal polyp in a dasatinib-treated patient—atypical in the general population; a contribution of dasatinib is possible but unproven.

**How commonly are dasatinib-associated colitis and non-colitis bleeding reported?**

- Dasatinib-associated colitis is far more frequently reported than non-colitis bleeding (33 vs. 3 published case reports) and often mimics inflammatory bowel disease both endoscopically and histologically.

**Do these gastrointestinal toxicities depend on leukaemia disease control?**

- Both bleeding and colitis phenotypes can arise independently of leukaemia disease control, including in patients with sustained deep molecular remission.

**What are the proposed mechanisms of dasatinib-associated gastrointestinal toxicities?**

- Proposed mechanisms include Src-family-kinase-mediated platelet dysfunction and endothelial barrier disruption (largely preclinical evidence), while colitis appears associated with T-cell-mediated immune activation.

**What are the risk factors for dasatinib-associated bleeding?**

- Thrombocytopenia and advanced-phase CML have the most support as independent risk factors for bleeding, largely from a single cohort; older age and higher or more frequent dosing show associations in the literature but have not been confirmed as independent predictors.

**How do we diagnose dasatinib-associated gastrointestinal toxicities?**

- No validated diagnostic criteria exist for dasatinib-induced GI toxicity; diagnosis relies on temporal association with drug exposure, exclusion of alternative causes, and reversibility with dose modification.

**How do we manage dasatinib-associated gastrointestinal toxicities?**

- Management should be individualised and multidisciplinary; dose reduction or temporary interruption are commonly used before switching TKI, but their effect on leukaemia control has not been systematically reported.

**Abstract:**

Dasatinib, an oral tyrosine kinase inhibitor used for chronic myeloid leukaemia (CML), is increasingly linked to gastrointestinal (GI) toxicities that remain under-recognised in clinical practice, ranging from mild, self-limiting symptoms to clinically significant GI bleeding and inflammatory colitis. This narrative review synthesises the published literature on dasatinib-associated GI bleeding and colitis, examining the mechanisms involved, including Src-family-kinase-mediated platelet dysfunction, endothelial barrier disruption and immune-driven T-cell infiltration, together with evidence on diagnosis and management. Dasatinib-associated colitis is far more extensively reported than non-colitis bleeding, has been reported across a broad adult age range and in both sexes, and has onset ranging from immediate to 9 years after initiation. Haematochezia, diarrhoea and abdominal pain are the most common presentations; endoscopic and histopathological findings are typically nonspecific and often mimic inflammatory bowel disease. The review is illustrated by two cases from a tertiary referral centre: delayed bleeding after cold-snare polypectomy of a diminutive 3 mm colorectal polyp, and chronic active colitis on combination asciminib–dasatinib therapy; formal causality assessment rated a dasatinib contribution as possible and probable, respectively. Dasatinib-associated GI complications, from delayed mucosal bleeding to chronic colitis, are clinically significant and warrant early recognition and multidisciplinary, individualised management. Standardised diagnostic criteria and peri-procedural guidance are needed to better balance bleeding risk against leukaemia control.

## 1. Introduction

Dasatinib, an oral second-generation tyrosine kinase inhibitor (TKI), is widely used in chronic myeloid leukaemia (CML) for its potent anti-leukaemic efficacy and favourable long-term outcomes [1,2]. Although generally well tolerated, dasatinib is increasingly linked to off-target gastrointestinal (GI) toxicities that remain under-recognised in routine clinical practice [3,4,5]. Common adverse effects include diarrhoea, nausea and dyspepsia; less frequent but clinically important complications include GI bleeding, haemorrhagic enterocolitis, and inflammatory bowel disease (IBD)-like colitis, occasionally complicated by cytomegalovirus infection. These manifestations are thought to reflect dasatinib’s effects on platelet function, endothelial integrity, immune regulation and mucosal homeostasis [5,6,7,8]. GI bleeding and inflammatory colitis are of particular clinical importance, carrying risks of significant morbidity, hospitalisation, interruption of anti-leukaemic therapy, and diagnostic uncertainty given their resemblance to other GI disorders [5,9,10]. No validated diagnostic criteria exist for dasatinib-induced GI toxicity; diagnosis remains clinical, based on temporal association with drug exposure, exclusion of alternative aetiologies, supportive endoscopic and histopathological findings, and improvement after withdrawal or dose reduction [5,10,11,12,13,14,15,16]. Despite growing case reports and pharmacovigilance data, the underlying mechanisms, true incidence, optimal diagnostic approach and management strategies remain incompletely defined. Existing reviews have addressed dasatinib’s broader adverse-reaction profile [3] or pooled gastrointestinal adverse events across multiple tyrosine kinase inhibitors [4], and single-phenotype case-based reviews and cohort studies have examined dasatinib-associated colitis in isolation [5,17]; to our knowledge, however, no prior work has directly contrasted the mechanistic basis, clinical phenotypes and management of dasatinib-associated bleeding and colitis side by side, and the absence of peri-procedural endoscopic guidance for dasatinib-treated patients has not been previously highlighted—gaps this review aims to address. This narrative review synthesises current evidence on dasatinib-associated GI bleeding and inflammatory colitis, complemented by two illustrative cases from institutional practice: clinically significant delayed post-polypectomy haemorrhage after cold snare resection of a diminutive colorectal polyp, and chronic active colitis associated with long-term dasatinib therapy. By integrating published evidence with real-world clinical experience, this review summarises pathogenesis, clinical presentation, diagnosis and management, and highlights practical considerations together with priorities for future research.

## 2. Methodology

### 2.1. Institutional Cases

Two illustrative cases, followed jointly by the Departments of Haematology and Gastroenterology, were identified during routine multidisciplinary care at Singapore General Hospital (SGH), a tertiary referral centre. Both patients were receiving dasatinib for CML and developed GI complications disproportionate to their underlying pathology, prompting consideration of dasatinib-associated toxicity. The first involved clinically significant delayed bleeding after cold snare resection of a diminutive (3 mm) colorectal polyp—exceptionally rare in the absence of conventional bleeding risk factors. The second presented with chronic inflammatory colitis whose clinical, endoscopic and histopathological features did not conform to a typical IBD phenotype and improved after dose reduction. Clinical data were retrospectively extracted from electronic medical records, and both cases were reviewed by the treating gastroenterologists, haematologists and pathologists to ensure clinicopathological correlation. Delayed post-polypectomy bleeding was defined according to the European Society of Gastrointestinal Endoscopy (ESGE) as bleeding that occurs after the end of the procedure and up to 30 days post-polypectomy and results in emergency attendance, hospitalisation or re-intervention [18]. The probability that dasatinib contributed to each event was assessed using the Naranjo adverse drug reaction probability scale [19] and the WHO–Uppsala Monitoring Centre (WHO-UMC) causality categories [20].

### 2.2. Literature Review

**Figure A1:**
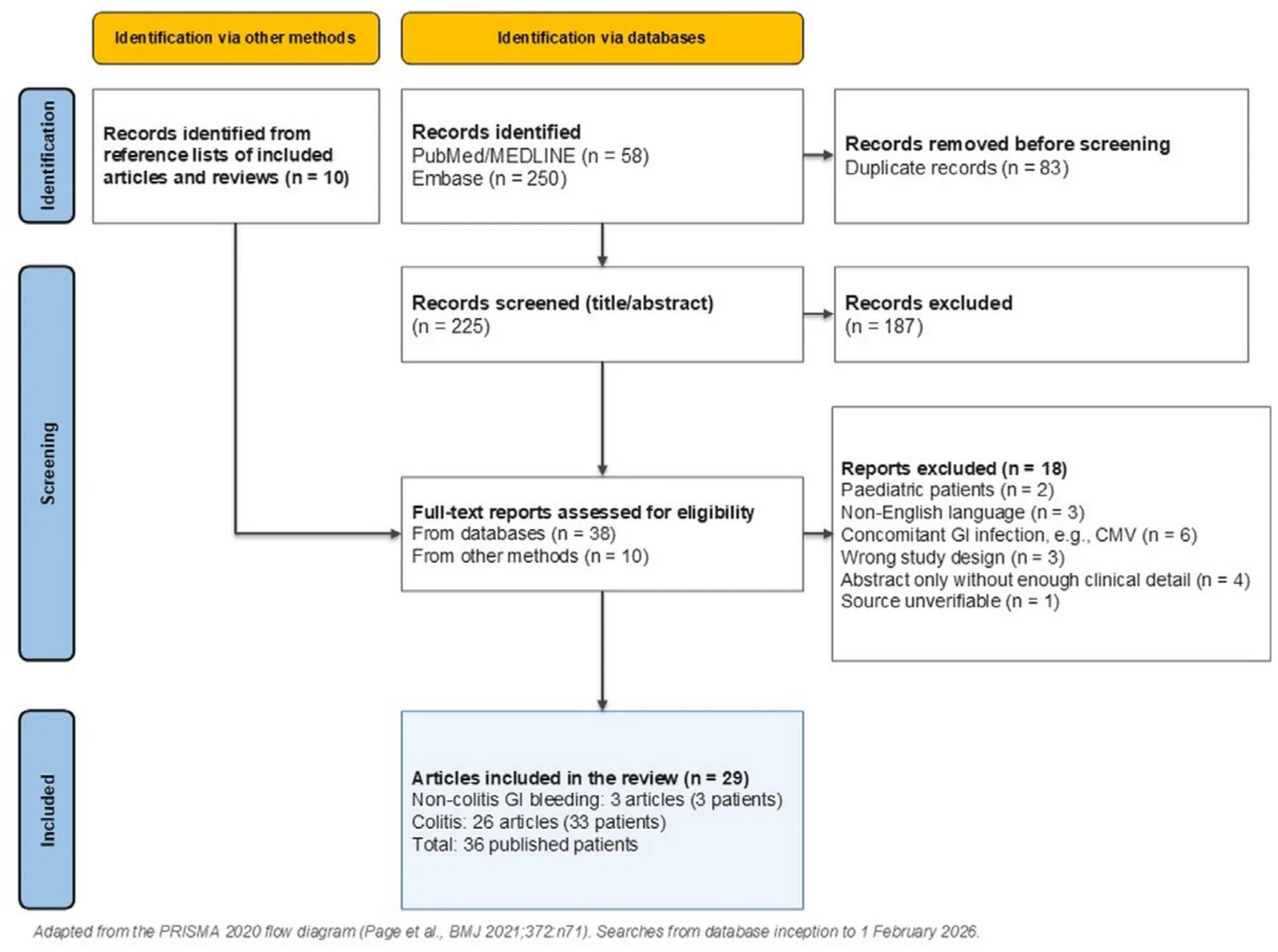
Flow diagram showing the search and selection process of the literature review, adapted from the PRISMA 2020 flow diagram [21].

An electronic literature search was conducted in PubMed and EMBASE from inception to 1 February 2026 using the search terms (hemorrhage OR bleeding OR gastrointestinal bleeding OR polypectomy OR post-polypectomy bleeding) AND (dasatinib), and (colitis OR drug-induced colitis OR drug-induced inflammation of bowel) AND (dasatinib), which together retrieved 308 records. The 308 records were imported into Covidence (Veritas Health Innovation, Melbourne, Australia; accessed on 4 October 2026), which removed 83 duplicate records (the same record retrieved in both databases or by both search strings); reports describing the same patient were identified by comparing patient characteristics and counted once. Titles, abstracts and full texts were screened by all authors. This review is narrative in design: the search was structured and is reported in a PRISMA 2020-style flow diagram [21] for transparency, but it was not conducted as a formal systematic review. Titles and abstracts were screened for relevance to dasatinib-associated gastrointestinal bleeding or colitis, and relevant articles were analysed in full (225 records were screened by title and abstract after duplicate removal, and 48 full-text reports were assessed, 38 from the databases and 10 from reference lists; Figure A1). Reports were eligible if they described individual adult patients with dasatinib-associated colitis or non-colitis GI bleeding; this yielded a final set of 3 articles reporting non-colitis GI bleeding and 26 articles reporting 33 individual colitis case reports (29 articles and 36 published patients in total; Figure A1), supplemented by manual review of reference lists of identified articles, which identified 10 further records for full-text assessment (Figure A1), as well as mechanistic, epidemiological and guideline literature. Several eligible reports were published as conference abstracts rather than full manuscripts; these are identified in the reference list and were included on the basis of the clinical detail available (four abstract-only reports were excluded; Figure A1), with awareness that abstract-only sources carry inherent reporting limitations and potential overlap with subsequent full publications that could not be exhaustively excluded. Reports involving concomitant infection were excluded to isolate cases attributable to dasatinib exposure alone, given that infection is itself a recognised cause of colitis and could confound attribution; paediatric populations and non-English-language publications were excluded as these were considered less representative of, or not directly generalisable to, typical adult dasatinib-treated patients in gastroenterology practice. After identifying all papers, study characteristics such as name of first author, baseline demographics of patients, indication, dose and duration of treatment, clinical presentation, endoscopic and histopathological findings as well as management and follow up were tabulated. Means, medians, interquartile ranges and percentages were calculated from the extracted case-level data solely to describe the published cases; because they derive from case reports, they are subject to publication and reporting bias and do not constitute epidemiological estimates.

## 3. Cases and Literature Findings

Two representative cases from our institution are presented to illustrate the diverse clinical manifestations and diagnostic challenges of dasatinib-induced gastrointestinal toxicities.

### 3.1. Illustrative Cases

#### 3.1.1. Case 1: Delayed Post-Polypectomy Bleeding in a Diminutive 3 mm Polyp

**Figure 1:**
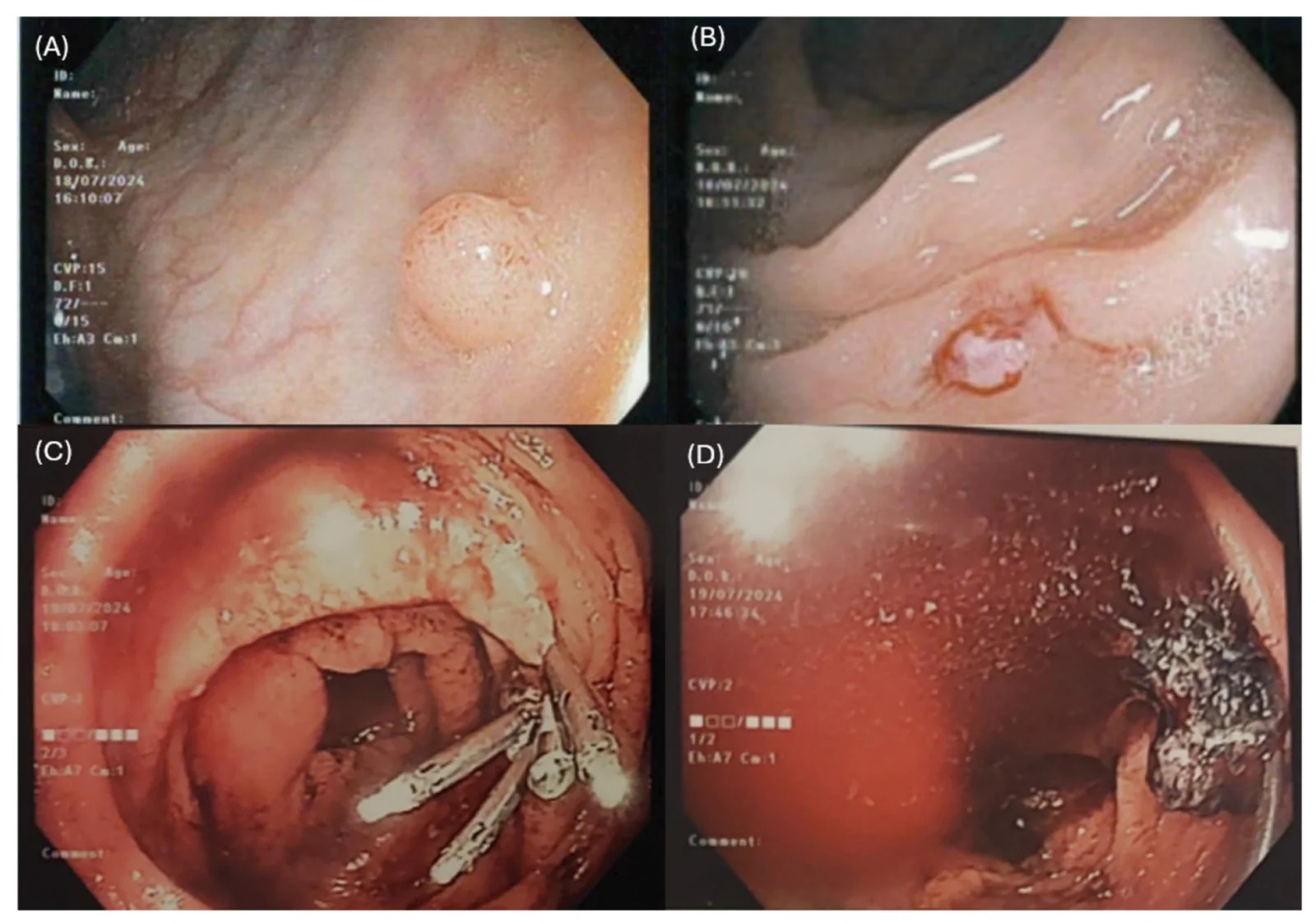
(**A**) Paris 1s 3 mm NICE II polyp prior to polypectomy on initial colonoscopy. (**B**) Polypectomy site immediately post polypectomy. (**C**) Previous polypectomy site with large blood clots and slow oozing on repeat colonoscopy. (**D**) Multiple clips placed subsequently and haemostasis secured.

A 72-year-old woman with CML in deep molecular remission (MR4.5) on dasatinib 100 mg daily presented with two weeks of dyspepsia and altered bowel habits. Colonoscopy revealed multiple small sessile polyps (3–5 mm) in the ascending, descending and sigmoid colon; polypectomies produced no immediate (intraprocedural) bleeding, and there was no visible vessel or mucosal injury at the polypectomy site. Approximately one hour later, she developed rectal bleeding but remained haemodynamically stable; digital rectal examination showed stale blood without active bleeding, and haemoglobin fell from 11.2 to 9.2 g/dL despite normal coagulation indices, platelet count and absence of drugs (including NSAIDs, antithrombotic) or comorbidities (including kidney or liver disease), which may increase bleeding risk. This met the European Society of Gastrointestinal Endoscopy (ESGE) definition of delayed post-polypectomy bleeding, defined as bleeding occurring after completion of the procedure and up to 30 days post-polypectomy that results in emergency attendance, hospitalisation, or the need for re-intervention [18]. Repeat same-day colonoscopy identified an overlying clot at the 3 mm ascending colon polypectomy site (Figure 1); cold snare polypectomy removed the clot, and four endoclips secured haemostasis, with no further bleeding sites identified; dasatinib was concurrently withheld for 48 h following haematology consultation, with no subsequent rebleeding, and dasatinib, which had been taken as usual on the day of the procedure, was restarted three days after the bleeding episode without further bleeding. No blood transfusion was required. Because endoscopic haemostasis and dasatinib withholding were instituted together, and no further polypectomy was performed after dasatinib was resumed, this case cannot distinguish the individual contribution of each intervention to the absence of further bleeding, nor does it constitute a valid rechallenge. Platelet function was not tested, so a dasatinib-related platelet defect could not be confirmed, and a causal role for dasatinib in this event remains uncertain (Table 1).

#### 3.1.2. Case 2: Dasatinib-Associated Chronic Active Colitis on Combination Asciminib–Dasatinib Therapy

**Figure 2:**
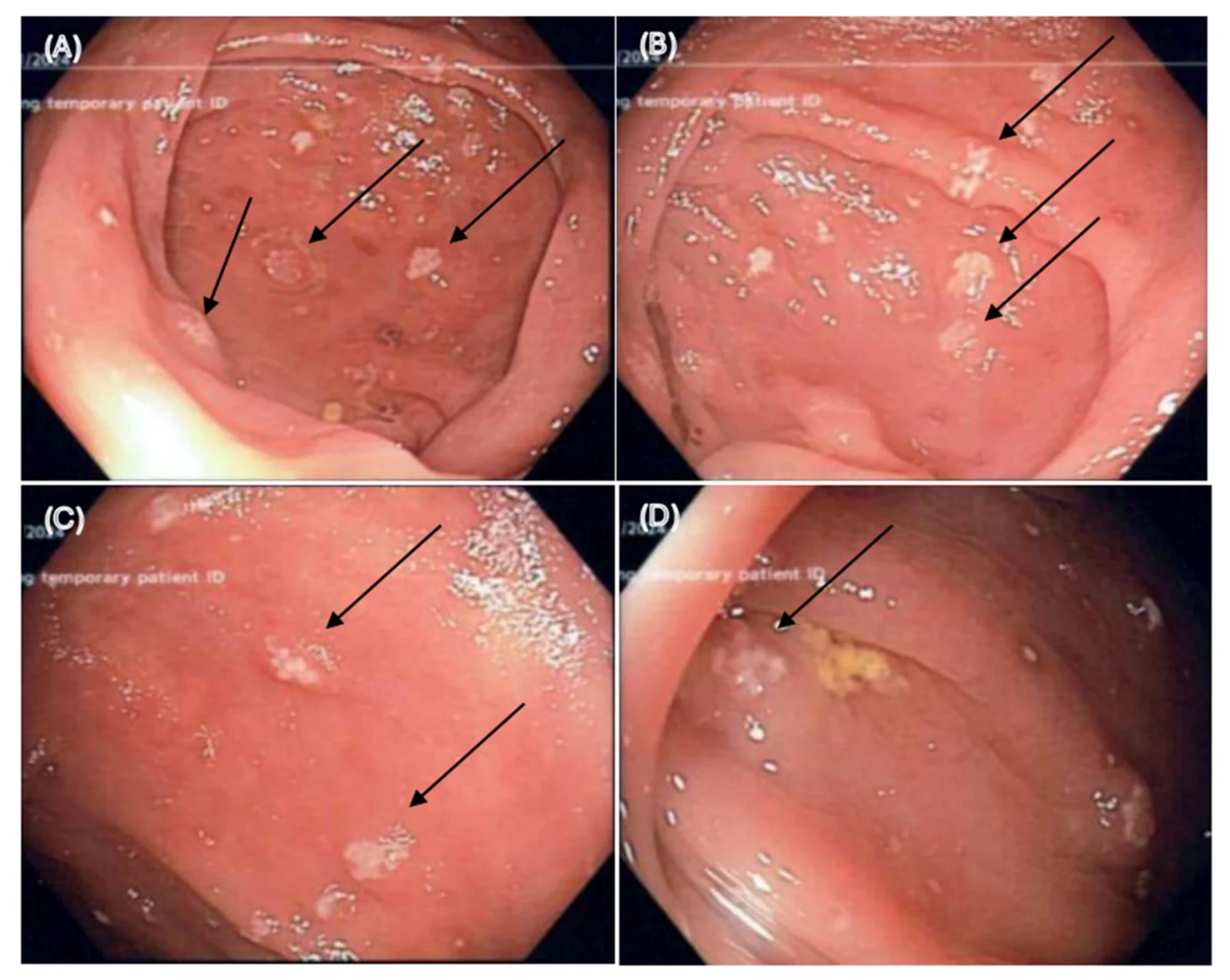
Endoscopic findings of dasatinib-associated chronic active colitis. (**A**) Caecal images, (**B**) ascending colon images, (**C**) descending Colon images, (**D**) sigmoid images, all showing multiple erosions with white exudates, erythema at the edges and loss of vascular marking in the surrounding mucosa. Biopsies were taken from these erosions for histopathology. Representative black arrows indicate some of these areas of erosion.

A 52-year-old man with CML on combination dasatinib–asciminib therapy presented with a one-year history of bloating, dyspepsia and six years of intermittent loose stools, recently worsening, without weight loss, haematochezia or melena. Review of his medication history revealed that the loose stools began with dasatinib initiation, which followed failure to achieve MMR on nilotinib. He subsequently achieved MMR after about 10 months of dasatinib and commenced asciminib on a clinical trial basis in combination with dasatinib about 2 years after starting dasatinib. Asciminib was given at 80 mg once daily in the morning within a phase I, multicentre, open-label study of oral asciminib (ABL001) in patients with CML or Ph+ ALL (CABL001X2101; ClinicalTrials.gov NCT02081378; institutional IRB reference 2018/2793), for which the patient gave written informed consent before starting treatment. Examination and laboratory findings, including platelet count and coagulation, were unremarkable. Stool culture, *Clostridioides difficile* tests and testing for ova and parasites were negative. C-reactive protein and faecal calprotectin were not measured, as the surveillance plan relied on endoscopy; the patient also missed multiple follow-up appointments, which accounts for the long interval between colonoscopies. Colonoscopy showed mild colitic changes from the descending colon to the sigmoid with relative rectal sparing, and multiple small sessile polypoidal lesions (<1 cm) with white exudates in the ascending colon and caecum; mucosa showed mild erythema and loss of vascular pattern without deep ulceration, bleeding or stricturing (Figure 2).

**Figure 3:**
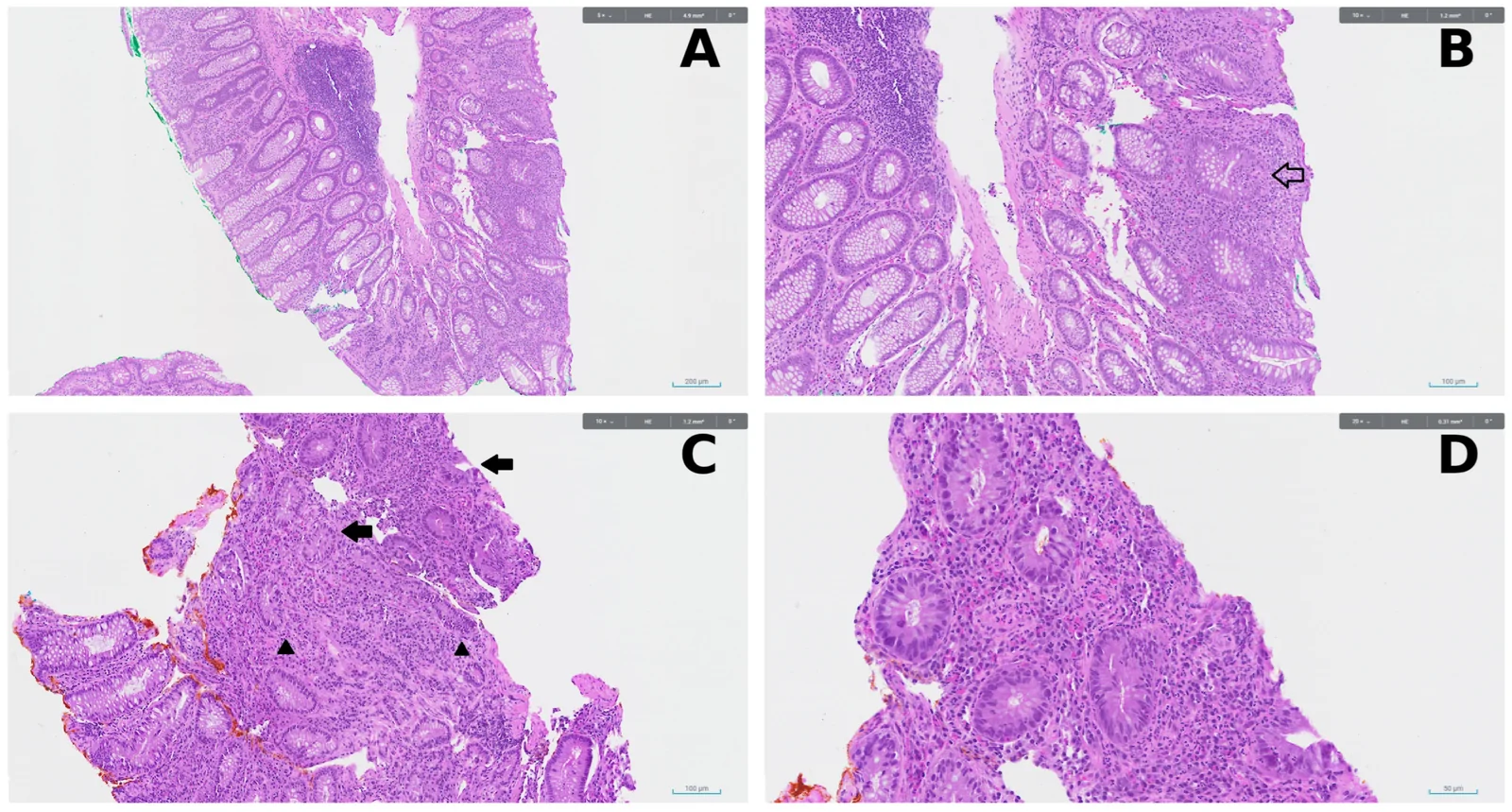
Histopathologic features of dasatinib-associated colitis. Mucosal fragment on H&E (**A**, 5×) shows a transition from preserved, orderly crypts on the left to irregular, distorted crypts on the right. H&E section (**B**, 10×) demonstrates active mucosal inflammation with scattered cryptitis and a focal crypt abscess (clear arrow) with surface erosions. Additional sections (**C**, 10×; **D**, 20×) highlight evidence of further cryptitis (solid arrows) with areas of crypt loss and dropout (area between triangles) and a lamina proprial mixed infiltrate of eosinophils, neutrophils and plasma cells.

Biopsies showed moderate active chronic colitis with patchy cryptitis, focal crypt abscesses, surface erosions, crypt architectural distortion and dropout, and a mixed eosinophil-, neutrophil- and plasma-cell-predominant infiltrate (Figure 3), without granulomas, dysplasia or malignancy; Grocott and Ziehl–Neelsen staining and immunostaining for cytomegalovirus were negative.

**Figure 4:**
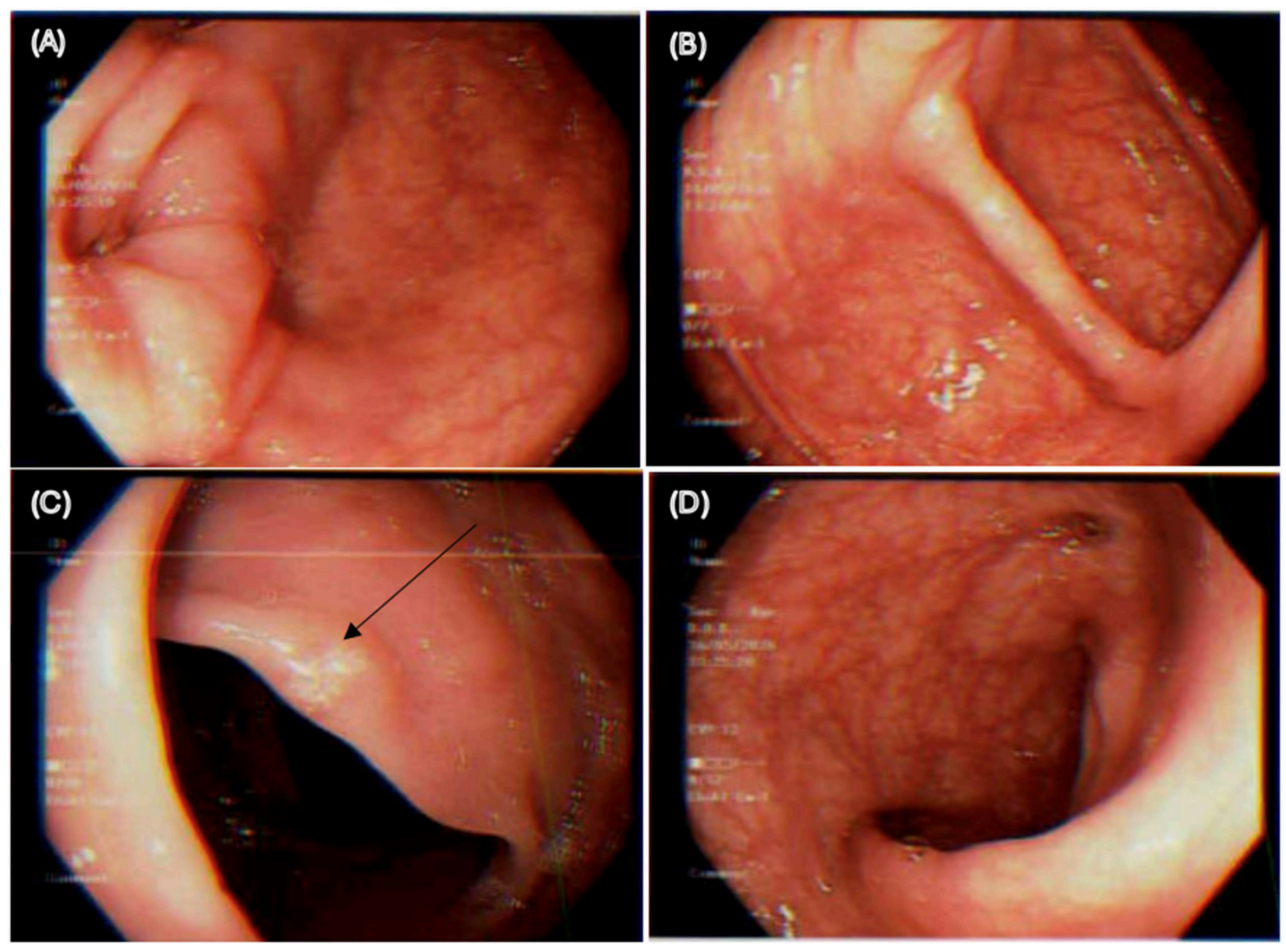
Endoscopic findings after reduction of Dasatinib dose. (**A**) Caecal images, (**B**) ascending colon images, (**C**) descending Colon images, (**D**) sigmoid images showing resolution of majority of previous erosions and restoration of vascular marking in the surrounding mucosa. Single erosion seen in the descending colon marked by arrow.

Given the temporal association and symptomatic improvement with dasatinib dose reduction (100 mg > 70 mg after about 7 years of dasatinib therapy, then >50 mg daily 2 months later) while asciminib remained unchanged, a diagnosis of dasatinib-associated colitis was made, although a contribution from asciminib could not be formally excluded (Table 1). Subsequent colonoscopy 18 months from initial colonoscopy showed endoscopic improvement of colitis (Figure 4). He was asymptomatic at the second colonoscopy, but dasatinib was subsequently stopped because mildly persistent colitis remained, although significantly improved; the patient remained well with no GI symptoms.

#### 3.1.3. Causality Assessment of the Institutional Cases

Causality was assessed for both cases using the Naranjo scale [19] and the WHO-UMC categories [20] (Table 1). For Case 1, the Naranjo score was 3 (possible) and the WHO-UMC category was possible: the temporal relationship was reasonable and the bleeding was objectively confirmed, but polypectomy is itself a sufficient cause of bleeding, dechallenge was confounded by concurrent endoscopic haemostasis, and no valid rechallenge occurred. For Case 2, the Naranjo score was 5 (probable, at the lower boundary) and the WHO-UMC category was probable/likely (borderline with possible): symptoms began with dasatinib initiation and preceded the introduction of asciminib, infection was excluded, endoscopic and histological abnormalities were objectively documented, and symptoms improved with stepwise dasatinib dose reduction while asciminib was unchanged; however, dasatinib was not rechallenged, no baseline endoscopy was available, and a contribution from asciminib cannot be completely excluded. The scores are sensitive to these judgements: scoring alternative causes as present in Case 2 (−1) lowers the Naranjo score to 4 (possible), whereas scoring them as excluded (+2) raises it to 7 (probable); for Case 1, scoring dechallenge as positive (+1) and alternative causes as absent (0) would raise the score to 5 (probable). Both instruments rely on discontinuation and rechallenge concepts that map imperfectly onto dose reduction and combination therapy, and neither can establish causation; the results are best read as supporting a possible (Case 1) and probable (Case 2) contribution of dasatinib rather than proof of causality.

### 3.2. Literature Review Findings

The published literature describes a clinically diverse spectrum of dasatinib-induced gastrointestinal toxicities, and our review summarises characteristics of individual published case reports and case series. However, these data are inherently subject to publication and reporting bias, including the preferential publication of unusual or diagnostically challenging cases and incomplete reporting of clinical variables. They are descriptive summaries rather than epidemiological estimates and should not be interpreted as estimates of the incidence, prevalence or risk.

The literature on dasatinib-associated non-colitis GI bleeding is limited, with only three case reports (Table A1); all describe clinically significant bleeding requiring multiple blood transfusions [7,22,23]. Endoscopic findings included angioectatic lesions and gastric antral vascular ectasia, both classically amenable to endoscopic haemostatic therapy, yet management in all three reports relied on dasatinib cessation (with or without a switch in TKI) rather than endoscopic intervention, with bleeding resolution in each case.

#### 3.2.1. Demographics of Reported Colitis Cases

Dasatinib-associated colitis, by contrast, has been more extensively reported: 33 published cases across 26 articles (Table 2, Table A2). Patients ranged from 18 to 82 years (mean 49 years among 28 cases with an exact age reported); 17 of 31 cases with reported sex were male (54.8%). Dasatinib was prescribed for CML in 26 cases (78.8%) and ALL in seven (21.2%) [10,15,24]. Reported doses at presentation ranged from 70–140 mg daily. Among 27 cases with a numerical treatment duration not restricted to time after dose escalation, the median reported duration was 144 days (IQR 51–365), with reported values ranging from 0 days to 9 years. The illustrative cases are excluded from these summaries.

**Table 2:** Demographic and clinical characteristics of 33 published cases of dasatinib-associated colitis across 26 articles.

Characteristic	Category	Value
Age, mean		49 years (*n* = 28)
Sex (*n* = 31)	Male	17/31 (54.8%)
	Female	14/31 (45.2%)
Indication for dasatinib (*n* = 33)	CML	26/33 (78.8%)
	ALL	7/33 (21.2%)
Reported CML disease phase (*n* = 13)	Chronic phase	5/13 (38.5%)
	Accelerated phase	4/13 (30.8%)
	Blast phase	4/13 (30.8%)
Reported CML response status (*n* = 8)	Haematologic remission	1/8 (12.5%)
	MMR	6/8 (75.0%)
	DMR	1/8 (12.5%)
Reported ALL treatment status (*n* = 5)	Relapse	2/5 (40.0%)
	Maintenance phase	3/5 (60.0%)
Dose at presentation (*n* = 19)	70 mg/day	4/19 (21.1%)
	100 mg/day	11/19 (57.9%)
	140 mg/day	4/19 (21.1%)
Reported treatment duration, median (IQR) (*n* = 27)		144 days (51–365)
Reported clinical presentation (*n* = 33)	Haematochezia	21/33 (63.6%)
	Diarrhoea	22/33 (66.7%)
	Abdominal pain	7/33 (21.2%)
	Bloating/distension	2/33 (6.1%)
	Weight loss	2/33 (6.1%)
	Fever	1/33 (3.0%)
	No GI symptoms	3/33 (9.1%)

Values are *n*/*N* (%) unless stated otherwise. The two institutional illustrative cases are excluded. Exact age was unavailable in five cases and sex in two; age ranges were not imputed. CML disease phase and response status are summarised separately where explicitly reported and may refer to overlapping patients or different clinical time points. Dose refers to the reported dose at presentation, before management of colitis. Duration excludes five unspecified values and the Yim et al. [25] interval measured only after dose escalation; the reported zero-day value is retained. Quartiles use linear interpolation. Clinical features may overlap; percentages describe documented features among all 33 cases, and non-reporting does not establish absence. “No GI symptoms” includes cases investigated for anaemia or occult blood positivity. CML, chronic myeloid leukaemia; ALL, acute lymphoblastic leukaemia; MMR, major molecular response; DMR, deep molecular response; IQR, interquartile range. These are characteristics of published case reports, are subject to publication and reporting bias, and do not represent epidemiological estimates.

#### 3.2.2. Clinical Presentation

Haematochezia, diarrhoea and abdominal pain were the most common presentations [8,10,13,14,15,16,17,24,25,26,27,28,29,30,31,32,33,34,35,36,37]; systemic features such as fever and weight loss were less frequent, and three cases lacked gastrointestinal symptoms despite iron deficiency anaemia or a positive faecal occult blood test [9,12,38].

#### 3.2.3. Endoscopic Findings

Endoscopic findings typically show diffuse or segmental mucosal erythema, erosions and superficial ulcerations [9,13,15,24,25,26,29,30,33,36,37,38], occasionally mimicking ulcerative or infectious colitis [5,7,10,38]; multiple small erosions predominate in the transverse and descending colon in up to 83% of cases [5,13,16,38], with nodular inflammatory lesions also reported [12,14,17,27,35,36].

#### 3.2.4. Histopathological Findings

Histologically, chronic active colitis with crypt architectural distortion [15,34], cryptitis [8,9,11,12,13,14,15,37] and inflammatory infiltration [8,9,11,12,13,15,24,25,27,31,34,37] are commonly described, often overlapping with inflammatory bowel disease, although granulomas are typically absent [5,7,38].

## 4. Discussion

Dasatinib-associated GI toxicity extends beyond common self-limiting symptoms to include clinically significant bleeding and inflammatory colitis. Although these complications likely arise through distinct pathways, they share overlapping mechanisms involving platelet dysfunction, endothelial barrier disruption and immune dysregulation, providing a framework for understanding which patients are at risk, how these complications present, and how they should be diagnosed and managed.

### 4.1. Proposed Pathophysiology

**Figure 5:**
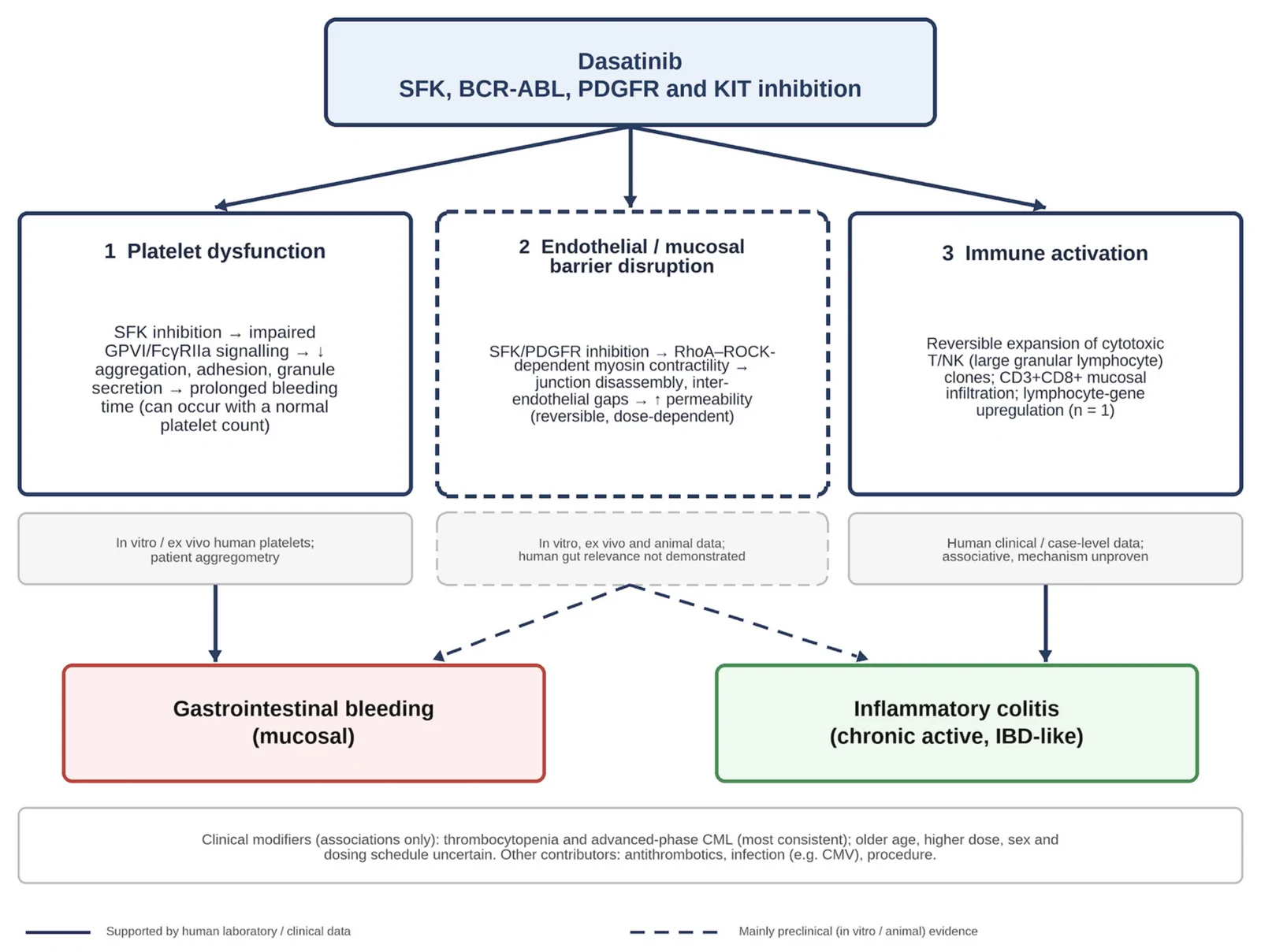
Proposed mechanisms linking dasatinib to gastrointestinal bleeding and colitis. Solid borders indicate mechanisms supported by human laboratory or clinical data; dashed borders indicate mechanisms supported mainly by in vitro, ex vivo or animal data, whose relevance to the human gut has not been demonstrated. Mechanisms were not directly assessed in the institutional cases. SFK, Src-family kinases; PDGFR, platelet-derived growth factor receptor; GPVI, glycoprotein VI; ROCK, Rho-associated kinase; NK, natural killer.

Dasatinib is proposed to predispose to bleeding through both platelet dysfunction and disruption of the GI endothelial barrier. Platelet dysfunction can occur even with normal platelet counts [22,39,40]: in vitro and ex vivo human platelet studies show dasatinib inhibits Src family kinases (SFKs), are central to platelet activation via GPVI/FcR and FcγRIIa signalling, impairing aggregation, adhesion and secretion [41,42,43], proposed to produce defective platelet plug formation and prolonged bleeding time in vivo. In vitro, ex vivo and animal studies further indicate that dasatinib disrupts endothelial barrier integrity through inhibition of SFKs and PDGFR, causing cytoskeletal contraction, disassembly of cell–cell junctions and inter-endothelial gap formation [44,45,46], producing a dose-dependent, reversible increase in permeability in these preclinical models [47,48,49]; whether this mechanism directly translates to mucosal barrier weakening and GI bleeding in patients has not been demonstrated and remains a proposed, rather than confirmed, clinical mechanism. Dasatinib-induced colitis, in contrast, appears to result from immune-driven inflammation with T-cell infiltration as a central mechanism: dasatinib is linked to reversible immune activation with clonal large granular lymphocyte expansion, including cytotoxic T- or NK-cell populations, correlating with inflammatory toxicities such as colitis [50], and transcriptomic analysis in a single reported patient showed upregulation of lymphocyte-related genes, consistent with, though not conclusive proof of, lymphocyte-mediated inflammation as a driver [9]. Endothelial barrier disruption may also contribute to colitis-related pathology. These proposed mechanisms are summarised in Figure 5; none was directly assessed in the institutional cases.

### 4.2. Risk Factors

Not all patients receiving dasatinib develop clinically significant GI toxicity; several patient- and treatment-related factors modify this risk. Bleeding episodes occur in up to 24% of patients in some cohorts, although most studies cite lower rates of 7–12% [40], with the GI tract the most frequently affected site (~81% of bleeding events) and lower GI bleeds more common than upper GI bleeds when including colitis-related manifestations [40]. Bleeding after resection of diminutive polyps is otherwise extremely rare: clinically significant delayed bleeding after cold snare polypectomy (CSP) of diminutive polyps occurs at rates typically <0.5%, often approaching zero in large series [51,52,53,54], and CSP remains the preferred technique for diminutive (≤5 mm) polyps given its favourable safety profile [18]. Most reported bleeding events instead occur with larger polyps (≥10 mm) [55,56,57], in patients on anticoagulants or antiplatelets [52,53,54,58], or when electrocautery is used [59,60,61]—making the haemoglobin drop and need for repeat endoscopic haemostasis after CSP of a 3 mm polyp in the case described in this review highly atypical; whether dasatinib contributed to this event cannot be determined from a single case. Reported risk factors for dasatinib-associated GI bleeding include age ≥ 60 years [6], thrombocytopenia and advanced disease phase [9,12,32,34]. The role of sex is less certain: in a Japanese spontaneous-report database, male sex was associated with GI bleeding reports overall (adjusted reporting odds ratio 1.47), whereas the association between dasatinib and GI bleeding was modified by age ≥ 60 years and female sex in interaction analysis [6], so female sex cannot be regarded as an established principal risk factor. If a true female predisposition exists, one possible explanation is higher drug exposure in women, as sex-related pharmacokinetic differences predict more adverse drug reactions in women across many drugs [62], although sex-specific dasatinib pharmacokinetic data are lacking; confounding and differential reporting are equally plausible, and these explanations remain speculative. The patient in Case 1 was a woman aged 72 years, but a single observation cannot test either association. In an observational cohort, 31 of 37 bleeding episodes occurred during treatment with dasatinib doses ≥140 mg/day [40]. This distribution should not be interpreted as a dose-specific incidence estimate, particularly because dose and disease phase were associated. Thrombocytopenia and advanced-phase CML were independently associated with bleeding, whereas dasatinib dose was not identified as an independent risk factor. A non-significant trend towards more bleeding with twice-daily than once-daily dosing was observed (*p* = 0.17) [40]. Overall, the evidence is strongest for thrombocytopenia and advanced-phase CML as independent bleeding risk factors in this cohort; associations with age, sex and treatment exposure require interpretation in the context of study design and potential confounding. Clinically significant bleeding may nevertheless occur without conventional procedural risk factors.

### 4.3. Dasatinib-Specific Toxicity or TKI Class Effect?

Whether these toxicities are specific to dasatinib or reflect a wider BCR-ABL TKI class effect is incompletely resolved. GI adverse events as a whole are common across the class: a meta-analysis of 43 studies in 10,769 patients with CML found diarrhoea to be the most frequent GI event (pooled 22.5%), with the highest mean GI adverse-event incidence for bosutinib (52.9%, including diarrhoea in 79.2%), followed by imatinib (24.2%), dasatinib (20.4%) and nilotinib (9.1%), and pooled GI haemorrhage of 3.5% [4]; these data do not separate colitis from non-inflammatory diarrhoea or attribute bleeding by agent. For bleeding, dasatinib appears to carry the most consistent signal. In a Japanese spontaneous-report database, significant GI bleeding signals were detected for dasatinib (adjusted reporting odds ratio 8.02) and, to a lesser degree, imatinib (1.81) [6]. Side-by-side laboratory studies likewise show that dasatinib consistently inhibits platelet function, whereas the effects of bosutinib, nilotinib and ponatinib are less consistent [63]; in a small ex vivo series of patients with CML, impaired platelet aggregation was common with dasatinib and was also seen, to a lesser extent, with imatinib, whereas most patients receiving nilotinib or bosutinib had normal aggregation [64]. Isolated reports nonetheless describe bleeding lesions and colitis with other TKIs, including resolution of imatinib-associated gastric antral vascular ectasia after drug cessation [65] and ulcerative colitis attributed to both imatinib and dasatinib [66], and TKI-induced colitis has been described more generally [24]. Taken together, GI bleeding and colitis appear most strongly and consistently associated with dasatinib, plausibly reflecting its potent Src-family kinase inhibition, but a contribution of shared off-target effects across the class cannot be excluded. Pharmacovigilance disproportionality and case reports cannot establish relative incidence, and cross-agent comparisons are confounded by differences in indication, line of therapy, disease phase and duration of exposure.

### 4.4. Diagnosis of Dasatinib-Related GI Toxicities

No validated diagnostic criteria exist for dasatinib-induced GI toxicity. Diagnosis relies on multifactorial clinical assessment: temporal association with drug exposure, reversibility upon dose reduction or withdrawal, exclusion of alternative aetiologies, and supportive endoscopic or histopathologic evidence [5,7,10]. Lacking a standardised scoring system specific to TKI-induced GI toxicity, generalised drug-reaction frameworks such as the modified Naranjo scale or WHO–Uppsala Monitoring Centre criteria are often applied, classifying events as ‘possible’, ‘probable’ or ‘definite’ based on clinical chronology and dechallenge–rechallenge outcomes [16,38]. Applied to the two institutional cases (Table 1), these frameworks rated a dasatinib contribution as possible in Case 1 and probable in Case 2, illustrating both their utility and their dependence on subjective judgements about alternative causes and dechallenge. In one retrospective single-centre cohort, colitis was diagnosed in 9 of 51 dasatinib-treated patients (17.6%) [5], a figure that should not be generalised as a population-wide prevalence, yet confirming the diagnosis requires a high index of suspicion, as presentations range from subclinical colitis found incidentally during anaemia workup to acute haemorrhagic colitis, and course from weeks-to-months of onset to a chronic, IBD-mimicking picture over years. Abdominal pain, diarrhoea and haematochezia are the most frequent symptoms [12,24]. Endoscopic and histopathological findings are heterogeneous and largely nonspecific, frequently mimicking inflammatory bowel disease and making clinicopathological correlation essential: colonoscopy typically shows nonspecific colitis with polypoidal lesions, while histology shows chronic active inflammation, cryptitis, microgranulomas and increased lamina propria eosinophils consistent with an immune-mediated pattern [15], with biopsies commonly showing dense CD3^+^ CD8^+^ T-cell infiltration [9,17]. Infective causes must be excluded; notably, Yamauchi et al. [5] identified evidence of cytomegalovirus reactivation, including nuclear inclusion bodies and positive immunostaining, among colonoscopically evaluated patients with dasatinib-induced colitis in their series, suggesting a possible CMV correlation; the precise proportion of CMV-positive cases and the small overall sample size limit causal inference. Symptomatic or endoscopic improvement after dose reduction or withdrawal strongly supports the diagnosis. Dasatinib has been associated with GI haemorrhage and colitis despite stable disease control [3,4,9], indicating toxicity can occur independently of leukaemia activity; ongoing vigilance for new-onset diarrhoea, bleeding or abdominal pain is warranted regardless of remission status or disease phase.

### 4.5. Guidance on Management of Dasatinib-Induced GI Toxicities

No disease-specific guidelines exist for managing dasatinib-associated GI toxicity. Management should reflect the severity of GI involvement, haematological status, and the need to maintain leukaemia control, with a cornerstone of maintaining a high index of suspicion, excluding alternative aetiologies, multidisciplinary discussion between gastroenterologists and haematologists, and individualised decisions on continuing, reducing or interrupting dasatinib. For GI bleeding, available evidence, largely from case reports and expert opinion, suggests that temporary interruption or dose reduction while avoiding concomitant anticoagulants or antiplatelets where possible may be reasonable [67]; by extension, similar measures could be considered after post-polypectomy bleeding, though guidance specific to planned mucosal interventions is lacking, and whether dasatinib should be held peri-procedurally or prophylactic clipping routinely employed is unknown. The joint BSG/ESGE guideline on endoscopy in patients on antiplatelet or anticoagulant therapy classifies polypectomy as a high-risk procedure for bleeding and bases periprocedural recommendations on the balance between thrombotic and haemorrhagic risk according to drug class [68]; however, this framework is built around conventional antiplatelet and anticoagulant agents and does not account for TKI-associated pharmacodynamic platelet dysfunction, so dasatinib-treated patients are not explicitly captured by current periprocedural risk stratification, and the magnitude of any additional dasatinib-attributable procedural bleeding risk relative to conventional antiplatelet or anticoagulant regimens remains unquantified. Because these recommendations cannot be extrapolated directly to patients receiving dasatinib, the peri-procedural approach in such patients should be individualised through discussion between the endoscopist and haematologist; whether dasatinib should be held, and for how long, is unknown, and interruption carries its own risk of loss of leukaemia control. The risk of recurrent haemorrhage must also be weighed against the risk of molecular relapse with cessation; the STOP 2G-TKI study, which evaluated planned discontinuation in patients with sustained deep molecular response, showed that a substantial proportion of such patients can achieve treatment-free remission with careful monitoring and no major safety concerns [69]—a different population and rationale from reactive interruption prompted by GI toxicity. Switching to an alternative TKI such as imatinib, which carries a lower disproportionality signal for GI bleeding in pharmacovigilance reporting data (adjusted reporting odds ratio 1.81 vs. 8.02 for dasatinib [6], a measure of relative reporting frequency rather than a direct incidence comparison), may be a reasonable alternative strategy in selected patients. Prophylactic clipping has been shown to reduce delayed bleeding after resection of large colonic polyps in randomised trial data [70], but has not been specifically evaluated for diminutive (<5 mm) polyps, whose baseline bleeding risk is inherently low [51,52,53,54]; there is therefore no evidence to support routine clipping of diminutive polyps, and whether clipping might be considered in dasatinib-treated patients with additional bleeding risk factors or larger polyps is unknown and can be regarded only as a possible consideration.

Management principles for dasatinib-associated colitis largely parallel those for bleeding: early recognition, individualised dose modification, and exclusion of alternative aetiologies (particularly infection) [10,71,72], given the condition’s generally idiosyncratic, reversible course. Initial treatment typically involves temporary cessation with corticosteroids in selected cases; three published cases continued or resumed dasatinib at a lower dose with reported clinical improvement [13,15,34], and the institutional Case 2 described in this review similarly showed symptomatic improvement after sequential dose reduction, though without a documented complete response, and dose reduction to 50 mg daily maintains efficacy with a more favourable overall GI safety profile in newly diagnosed CML-CP [73]—though this frontline population and endpoint differ from colitis-specific dose reduction in previously treated patients—although a clear dose–response relationship for colitis itself has not been established. Nine patients in the literature were transitioned to alternative therapies (eg. TKIs) [11,14,17,27,28,30,35,36,38], while asymptomatic patients tended to continue dasatinib at the same dose [9,12,16,37]; rechallenge after symptom resolution may be considered, reserving alternative TKIs for recurrence [27]. The decision to continue, reduce or switch therapy should reflect disease status and severity of GI involvement. However, the published cases did not systematically report leukaemia response after dose reduction, interruption or TKI switching, so the effect of these strategies on disease control could not be synthesised.

Prompt symptom-triggered evaluation of new-onset diarrhoea, bleeding or abdominal pain, and multidisciplinary coordination between haematology and gastroenterology remain essential to balance leukaemia control against GI morbidity [7,72]. Other measures—routine surveillance with inflammatory markers or faecal calprotectin, baseline colonoscopy before high-dose therapy, dietary modification and prophylactic endoscopic clipping—are supported by little or no dasatinib-specific evidence; they are presented here as possible considerations for individual patients, not as recommendations, and their benefit is unproven. Preclinical data suggest ruscogenin may enhance intestinal barrier function in this setting [48], though this remains experimental.

Evidence for prophylactic clipping is limited to large polyps: clip closure reduced delayed bleeding after resection of large (≥20 mm) non-pedunculated lesions, mainly in the proximal colon, in one randomised trial [70], but did not significantly reduce delayed bleeding after resection of polyps ≥ 10 mm in another [74]. Clipping has not been evaluated after resection of diminutive polyps, whose baseline bleeding risk is very low [51,52,53,54], or in dasatinib-treated patients. Routine clipping in this setting is therefore not supported by current evidence; it may be considered on an individual basis in dasatinib-treated patients with additional bleeding risk factors who undergo resection of larger lesions.

### 4.6. Limitations

Establishing a direct causal relationship between dasatinib and GI toxicity remains challenging: current evidence derives predominantly from case reports, small series and pharmacovigilance studies, which show consistent temporal association but cannot definitively distinguish direct induction of injury from potentiation of pre-existing mucosal vulnerability or procedural risk. Patients with CML or ALL commonly have complex underlying disease, prior treatment exposure, immunosuppression, multiple concomitant medications and several alternative causes of GI bleeding or colitis, so individual case reports—including our own—cannot establish dasatinib as the direct cause. No formal risk-of-bias appraisal (e.g., the Murad et al. [75] case-report assessment tool) was applied to the included case reports, nor was adherence to the CARE reporting checklist (which assesses completeness of reporting rather than methodological or causal validity) formally verified, and the pooled demographic and clinical percentages presented in this review should therefore be interpreted as descriptive summaries of heterogeneous, non-appraised sources rather than validated epidemiological estimates. They are further subject to publication and reporting bias, and the review was restricted to two databases (PubMed and EMBASE) and to English-language reports; exclusion of reports with concomitant infection, including cytomegalovirus, may also have under-represented infection-associated colitis. Heterogeneous presentations and the absence of validated diagnostic criteria contribute to variability in case definition, and diagnosis relies largely on clinicopathological correlation and exclusion of alternative aetiologies rather than objective biomarkers. The cases described also have inherent limitations: in Case 1, platelet function testing and assessment of Src-dependent signalling were not performed, precluding direct demonstration of qualitative platelet dysfunction; in Case 2, the absence of baseline endoscopy before dasatinib initiation and concurrent asciminib use, together with the absence of inflammatory markers (C-reactive protein and faecal calprotectin), limit definitive attribution; however, colitis has not been reported as a characteristic asciminib-associated toxicity in pivotal trials or the largest published real-world cohort (*n* = 77) [76], whose adverse-effect profile was dominated by cytopenias, arthralgia and pancreatic enzyme elevation rather than colonic inflammation; this cohort is nonetheless underpowered to exclude a rare asciminib-related or combination-therapy effect, so asciminib and a combined-agent contribution cannot be definitively excluded. The absence of a reported asciminib-colitis signal, together with the temporal relationship and symptomatic improvement with dasatinib dose reduction, favours but does not confirm a dasatinib-driven process. Leukaemia response after management of GI toxicity was not systematically reported in the reviewed cases, so the effect of dose reduction, interruption or TKI switching on disease control could not be assessed. Our discussion also centres on dasatinib, and a wider TKI class effect cannot be excluded. Priorities for addressing these gaps are outlined in the Future Directions section below. Overall, current evidence supports a possible—but not yet definitively established—association between dasatinib and GI toxicity; GI events in dasatinib-treated patients should be interpreted with caution, balancing bleeding or inflammatory risk against the need to maintain deep molecular remission.

## 5. Future Directions

Priorities for future research should first establish large, prospective, multicentre registries and pooled case series with a defined exposed denominator, incorporating symptom latency, endoscopic severity, histologic activity, temporal treatment response and subsequent leukaemia control, to define the true incidence and clinical phenotypes of dasatinib-associated bleeding and colitis—basic case definition and counting that current evidence cannot support. Building on this foundation, standardised, validated diagnostic criteria should be developed, incorporating objective measures such as platelet function assays, Src-family-kinase signalling assessment, pharmacokinetic monitoring, and biomarkers of endothelial and epithelial barrier dysfunction, alongside standardised histopathological reporting and prospective evaluation of mucosal healing after dose modification to strengthen causal inference and differentiate drug-induced colitis from inflammatory bowel disease or infectious colitis. Prospective procedural studies should then establish peri-procedural guidance specific to dasatinib-treated patients undergoing endoscopic interventions—including whether dasatinib should be held before polypectomy and the role of prophylactic clipping—as current antiplatelet/anticoagulant-focused frameworks do not account for TKI-associated pharmacodynamic platelet dysfunction. Finally, dedicated comparative studies of the gastrointestinal safety profiles of dasatinib against alternative BCR-ABL tyrosine kinase inhibitors would help inform individualised treatment selection in patients at elevated bleeding or colitis risk.

## 6. Conclusions

Dasatinib is associated with a spectrum of gastrointestinal toxicities, from mucosal bleeding to chronic inflammatory colitis, through proposed mechanisms—platelet dysfunction, endothelial disruption and immune-mediated mucosal injury—that carry differing levels of evidentiary support and were not directly demonstrated in either institutional case. Despite varying presentations, the available reports suggest common management principles, although no guideline exists: a high index of suspicion for early recognition, exclusion of alternative causes, and drug interruption or dose adjustment under close haematologic monitoring. Decisions should be individualised, balancing the risk and severity of gastrointestinal complications against the imperative to maintain leukaemia control—underscoring the need for vigilant, multidisciplinary care. Whether dose reduction, interruption or switching TKI preserves leukaemia control after GI toxicity has not been systematically reported and remains to be established.

## Figures and Tables

**Table 1:** Causality assessment of the two institutional cases using the Naranjo adverse drug reaction probability scale and the WHO-UMC categories.

Naranjo Item	Case 1	Case 2
1. Previous conclusive reports of this reaction?	+1	+1
2. Event appeared after the suspected drug was given?	+2	+2
3. Improved when the drug was discontinued or an antagonist given?	0 (haemostasis and withholding concurrent)	0 (improvement on dose reduction scored under item 8)
4. Reappeared when the drug was re-administered?	0 (resumed; no repeat polypectomy)	0 (not performed)
5. Alternative causes that could on their own have caused the reaction?	−1 (polypectomy wound)	0 (infection excluded; asciminib co-exposure and pre-existing IBD not fully excluded)
6. Reappeared when a placebo was given?	0	0
7. Drug detected in blood at toxic concentration?	0 (not measured)	0 (not measured)
8. Reaction less severe when the dose was reduced?	0	+1 (improved with 100→70→50 mg)
9. Similar reaction to the same or similar drug previously?	0	0
10. Confirmed by objective evidence?	+1 (clot at site; haemoglobin fall)	+1 (endoscopy and histology)
Naranjo total (category)	3 (possible)	5 (probable, lower boundary)
WHO-UMC category	Possible	Probable/likely (borderline with possible)

Naranjo categories: ≥9 definite; 5–8 probable; 1–4 possible; ≤0 doubtful. WHO-UMC, World Health Organization–Uppsala Monitoring Centre; IBD, inflammatory bowel disease.

## Data Availability

Data from this study is available upon reasonable request to the corresponding author.

## Abbreviations

The following abbreviations are used in this manuscript:

CML	Chronic myeloid leukaemia
TKI	Tyrosine kinase inhibitor
IBD	Inflammatory bowel disease
CSP	Cold snare polypectomy
CMV	Cytomegalovirus
BSG	British Society of Gastroenterology
ESGE	European Society of Gastrointestinal Endoscopy

## Appendix A

### Appendix A.2. Literature Review Findings

**Table A1:** Literature review—case reports of dasatinib-induced gastrointestinal bleeding.

Reference	Age/Sex	Indication of Dasatinib	Disease Phase	Dose	Duration of Treatment (Days)	Presentation	GI Adverse Effect	Endoscopic Findings	Treatment	Follow Up
Akhtar et al. [7]	57 Female	Ph+ B-cell ALL	n.s.	n.s.	30	Haematochezia	Small bowel bleeding	Small bowel enteroscopy: Friable mucosa in the duodenum and jejunum with no apparent inflammation and no distinctive lesions amenable to endoscopic treatment	Dasatinib ceased, switched to nilotinib	No further bleeding
Kostos et al. [22]	84 Female	CML	Chronic phase (MMR)	100 mg/day	150	Melena	GI bleeding	Capsule endoscopy: Active bleeding site associated with angioectactic lesions in the proximal jejunum	Dasatinib ceased	No further bleeding, switched to low-dose imatinib
Ohta et al. [23]	46 Male	Ph+ ALL	n.s.	n.s.	6	Melena	GAVE	OGD: Oozing from scattered mucosal erosions in the gastric antrum with a ‘watermelon appearance’	Dasatinib ceased	No further bleeding, endoscopic resolution
Case 1	72 Female	CML	Chronic Phase (DMR)	100 mg/day	1753	Haematochezia (post scopes)	Post-polypectomy bleeding	Initial colonoscopy: multiple small sessile polyps (3–5 mm) in the ascending, descending, and sigmoid colon and polypectomies were performed | Repeat colonoscopy: overlying clot at the 3 mm ascending colon polypectomy site	Dasatinib held off for 48 h	No further bleeding, Dasatinib restarted

**Table A2:** Literature review—case reports of dasatinib-induced colitis.

Reference	Age/Sex	Indication of Dasatinib	Disease Phase	Dose	Duration of Treatment (Days)	Clinical Presentation	GI Adverse Events	Endoscopic Findings on Diagnosis	Histopathology Findings on Diagnosis	Treatment	Follow Up
Ahn et al. [33]	68 Male	CML	Blast phase	140 mg/day	90	Diarrhoea, Haematochezia, Fever	Haemorrhagic colitis	Several mucosal hyperemia and edema with spontaneous bleeding were observed without definite ulceration or mass	Active Colitis	Dasatinib stopped and symptoms resolved	Dasatinib restarted after due to progression of CML
											Symptoms recurred; held off dasatinib indefinitely
Aldoss et al. [10]	47 Male	Ph+ ALL post transplant	Maintenance	n.s.	108	Haematochezia	Colitis	Inflammation	Focal, minimal active colitis	Dasatinib stopped, Budesonide	Symptoms resolved
	47 Male	Ph+ ALL post transplant	Relapse	n.s.	145	Diarrhoea	Colitis	Erythema and pseudo-membrane	No acute inflammation but stromal fibrosis and granulation tissue formation, with focal apoptosis	Dasatinib stopped, Budesonide	Dasatinib restarted; symptoms recurred, dasatinib stopped
	47 Male	Ph+ ALL post transplant	Maintenance	n.s.	92	Diarrhoea, Abdominal pain	Colitis	Inflammation	Mild acute colitis	Dasatinib stopped; Prednisone increased from 5 to 10 mg daily, budesonide was added	Dasatinib restarted. Symptoms recurred, dasatinib stopped
	50 Male	Ph+ ALL post transplant	Relapse	n.s.	132	Diarrhoea	Colitis	Mild inflammation and erythema, from proximal sigmoid to rectum	Questionable mild GVHD	Dasatinib stopped, Prednisone added	Dasatinib restarted. Symptoms recurred, dasatinib stopped and budesonide added
	27 Female	Ph+ ALL post transplant	Maintenance	n.s.	14	Haematochezia	Colitis	Mild inflammation, erythema	Focal hyperplastic changes	Dasatinib stopped, Added budesonide and prednisone	Symptoms resolved
Arborea et al. [37]	34 Male	CML	n.s.	100 mg/day	365	Diarrhoea	Colitis	Multiple shallow areas of mucosal erosion measuring 6–8 mm in the descending and sigmoid colon	Mild to moderate active inflammation with mild cryptitis; the inflammatory infiltrate was composed of a mixture of lymphocytes, plasma cells, neutrophils, and increased numbers of eosinophils	Dasatinib continued in absence of haematochezia or abdominal pain	n.s.
Chisti et al. [34]	47 Female	CML	Blast phase (MMR)	100 mg/day for 1 month, then 70 mg/day (due to non-GI side effects) for 10 months	330	Diarrhoea, Haematochezia	Haemorrhagic colitis	Patchy area of mildly congested, erythematous, granular and vascular patterned with decreased mucosa found from the rectum to the transverse colon	Crypt architectural changes, increased lymphoplasmacytic inflammation of the lamina propria and focal areas with increased intraepithelial lymphocytes	Dasatinib initially restarted at 50 mg/day but colitis recurred; course of steroids started for recurrent episode	Restarted at lower dose dasatinib (20 mg) with no further episodes
Contino et al. [14]	69 Male	CML	n.s.	n.s.	365	Diarrhoea, Abdominal pain, Anaemia	Crohn’s-like colitis	Colonoscopy: possible erythematous angioectasias in the ascending colon, and nodular erythematous mucosa with punctate white plaques in the descending and sigmoid colon	Surface erosion, acute cryptitis, and changes indicative of chronic injury	Stopped dasatinib, switched to imatinib	Symptoms resolved
								OGD: non-erosive gastritis and two small duodenal AVMs			
Demiröz et al. [26]	26 Male	Ph+ CML	n.s.	100 mg/day	1095	Diarrhoea, Abdominal distension, Fatigue	Colitis	Initially showed skip lesions with ulcerations, subsequently diffuse mucosal hyperemia and loss of vascular pattern in all colonic segments, with a normal terminal ileum	Dense neutrophilic infiltration in the lamina propria without crypt abscesses or distortion	Initially treated as per Crohn disease with budesonide and mesalamine	Symptoms persisted and dasatinib stopped
										Repeat colonoscopy done due to persistent symptoms—stopped dasatinib after	Symptoms resolved
Erkut et al. [35]	55	CML	Blast phase (haematologic recurrence)	100 mg/day	14	Haematochezia, Abdominal pain	Haemorrhagic colitis	No active bleeding, multiple millimetric nodular hyperemic lesions on the mucosa of left side of colon	Nonspecific active colitis	Dasatinib stopped	Dasatinib restarted after endoscopic resolution however colitis recurred; Dasatinib stopped again
											Switched to nilotinib 400 mg/day with no further complications
Eskazan et al. [36]	18 Male	CML	Chronic phase	100 mg/day	42	Diarrhoea, Haematochezia	Colitis	Exudation, erosions and multiple ulcers with nodular hyperemic lesions in the entire colon	Nonspecific colitis	Steroids and 5-amino-salicylate started, dasatinib stopped	Started on hydroxyurea, Symptoms resolved
Gluch et al. [11]	50s Female	CML	Not specified (MMR)	70 mg/day	3285	Haematochezia, Weight loss	Colitis	Moderate patchy inflammation in the sigmoid and descending colon	Mild active chronic inflammation with cryptitis, basal plasmacytosis and eosinophilia without intraepithelial lymphocytosis, granulomas, viral inclusions, parasites or dysplasia	Dasatinib stopped	Endoscopic and histologic resolution; switched to nilotinib
Grillo et al. [15]	50s Male	CML	n.s.	n.s.	n.s.	Diarrhoea, Haematochezia, Anaemia	Crohn’s-like colitis	Patchy erythema of the mucosa from the transverse colon to the rectum with multiple erosions and inflammatory-type polyps	Discontinuous, transmucosal chronic active inflammation with associated cryptitis as well as focal architectural crypt distortion	Initially treated for Crohn disease with sulfasalazine, stopped dasatinib after	Asymptomatic with endoscopic and histologic resolution
	mid-60s Male	CML	n.s.	n.s.	n.s.	Diarrhoea	Crohn’s-like colitis	Normal ileum, patchy hyperaemia in the left colon and rectum	Patchy chronic active transmucosal inflammation with focal erosions, occasional microgranulomas and mild crypt distortion	Dasatinib stopped	Symptoms resolved
	60s Male	CML	n.s.	n.s.	216	Diarrhoea, Abdominal bloating	Colitis	n.s.	Patchy chronic active inflammation with associated cryptitis and marked plasma cell infiltrate with Russel bodies and basal plasmocytosis	Dasatinib dose reduced and started mesalazine	Symptoms resolved
	n.s.	ALL	n.s.	n.s.	n.s.	Diarrhoea, Haematochezia	Crohn’s-like colitis	Erythematous sigmoid	Discontinuous chronic active inflammation with superficial erosions and basal plasmocytosis	Dasatinib stopped	Symptoms resolved
Jung et al. [24]	45 Female	Ph+ ALL	n.s.	100 mg/day	14	Diarrhoea, Haematochezia, Abdominal pain	Colitis	Numerous erosions/ulcers with spontaneous oozing, bleeding, and surface hemorrhagic clots, as well as many yellowish exudative plaques from the transverse colon to the rectum	Pseudomembranes from erupting crypts with heavy infiltration of eosinophils and neutrophils, as well as fibrin clots	Dasatinib stopped	Symptoms resolved with endoscopic resolution
Khalil et al. [13]	53 Female	CML	n.s.	n.s.	60	Haematochezia	Colitis	No active bleeding, scattered patchy erythema with focal erosions throughout the colon	Chronic active inflammation including cryptitis, crypt abscesses, and scattered eosinophils	Dasatinib dose reduced	Symptoms improved
Khurana et al. [38]	59 Male	CML	n.s.	100 mg/day	2555	Iron deficiency anaemia, asymptomatic	Colitis	Patchy areas of erosions and inflammation throughout the colon with rectosigmoid sparing and normal terminal ileum	Mild to moderate active and chronic colitis	Dasatinib stopped	Symptom and endoscopic resolution; switched to alternative therapy
Kmira et al. [27]	36 Female	CML	Blast Phase	140 mg/day	34	Diarrhoea, Haematochezia, Abdominal pain	Haemorrhagic colitis	No active bleeding, but there were multiple millimetric, nodular, hyperemic lesions on the mucosa involving the entire colon	Nonspecific colitis with a well-preserved crypt structure and lymphocytic infiltration in the lamina propria; infiltrative lymphocytes expressed a high proportion of CD3 and sparse of CD20	Broad spectrum antibiotics, hydration, dasatinib stopped	Dasatinib restarted however symptoms recurred
											Switched to nilotinib 800 mg/day
Maat et al. [28]	34 Female	CML	Chronic phase (MMR)	100 mg/day for 7 months, reduced to 70mg/day due to non-GI side effects	324	Diarrhoea, Haematochezia	Colitis	Patchy erythema and inflammation in the sigmoid colon with rectal sparing, and retroflexion revealed small anal papillae	Large bowel mucosal fragments with no significant pathological changes	Dasatinib stopped	Symptoms resolved; switched to imatinib
Muhammad et al. [29]	82 Male	CML	n.s.	n.s.	n.s.	Haematochezia	Colitis	Multiple shallow non-bleeding ulcers in the entire colon ranging in diameter from 2 mm to 10 mm	Patchy active colitis and ulcerations with features suggestive of drug toxicity	Initially started on budesonide however symptoms recurred	Ulcerations persisted during follow up colonoscopy, likely due to impaired mucosal healing with prednisone; endoscopic resolution noted after dasatinib and prednisone stopped
										Dasatinib and budesonide stopped, started on prednisone 40 mg/day	
Ono et al. [77]	43 Female	CML	Accelerated phase	140 mg/day	36	Diarrhoea, Melena	Haemorrhagic colonic ulcers	Multiple aphthous ulcers of the colon	n.s.	Dasatinib stopped	Symptoms resolved with endoscopic resolution; planned for allogeneic cord blood transplantation
Oshima et al. [9]	56 Female	CML	Accelerated phase (DMR)	100 mg/day	144	Faecal occult blood positive, Asymptomatic	Colitis	Multiple shallow areas of mucosal erosion with yellow exudate and congested erythematous lesions, which seemed to occur in a longitudinal direction from the transverse to descending colon	Increased lymphocytes infiltration to the lamina propria with cryptitis	Dasatinib continued as patient asymptomatic	Dasatinib reduced to 70 mg/daily due to non-GI side effects; endoscopic and histological improvements noted after
Patodi et al. [30]	59 Male	CML	Accelerated phase	70 mg/day	1095	Diarrhoea, Haematochezia, Weight loss	Haemorrhagic colitis	Granular and congested mucosa in the rectum along with large ulcers between the descending colon and splenic flexure	Acute colitis	Initially treated for ulcerative colitis with steroids and mesalazine	Symptoms recurred; started on steroids, stopped dasatinib, switched to nilotinib
											Symptoms resolved; endoscopic resolution with improvement histologically
Perdigoto et al. [16]	54 Male	CML	MMR	n.s.	900	Diarrhoea, Haematochezia	Colitis	Diffuse papular erythematous areas, more severe in the sigmoid colon	Nonspecific colitis	Dasatinib continued as symptoms spontaneously resolved	Symptoms resolved with endoscopic improvement
Shanshal et al. [17]	43 Female	CML	Chronic phase	100 mg/day	90	Haematochezia	Haemorrhagic colitis	Nodular patchy pattern of inflammation with a friable surface consistent with multinodular focal active colitis	Focally active colitis	Dasatinib stopped	Dasatinib restarted at 50 mg/day
											Symptoms recurred, switched to nilotinib 150 mg BD
Sunami et al. [31]	26 Female	CML	Chronic phase	n.s.	n.s.	Diarrhoea, Haematochezia	Haemorrhagic colitis	n.s.	CD8-positive T-lymphocyte infiltration into the colon mucosa	n.s.	n.s.
Tamilarasan et al. [12]	61 Female	CML	Chronic phase (MMR)	100 mg/day	270	Iron deficiency anaemia, asymptomatic	Colitis	Numerous, diminutive (<3 mm), pigmented nodules throughout the colon	Mild to moderate, active inflammation with cryptitis; the inflammatory infiltrate contained a mixture of lymphocytes, plasma cells, eosinophils and neutrophils; very occasional branched crypts and borderline basal plasmacytosis were also features	Dasatinib continued as patient asymptomatic and no clinically significant consequences	Remained asymptomatic and continued on dasatinib
Tozluklu et al. [8]	71 Female	CML	Haematologic remission	100 mg/day	0	Diarrhoea, Haematochezia, Abdominal pain	Crohn’s-like colitis	Minimal terminal ileal hyperemia with edematous and granular colonic mucosa	Chronic active colitis with cryptitis, terminal ileitis, and eosinophilic infiltration, but lacked granulomas or specific features diagnostic of IBD	Stopped dasatinib	Symptoms resolved
Wubiee et al. [32]	60 Female	CML	n.s.	100 mg/day	730	Diarrhoea, Haematochezia, Abdominal pain	Colitis	Diffuse colitis	n.s.	Started mesalamine but symptoms recurred intermittently	Persistent endoscopic findings of diffuse colitis
Yim et al. [25]	54 Male	CML	Accelerated phase (MMR)	100 mg/day for 3 years, increased to 140 mg/day due to haematologic recurrence	30 (days at higher dose)	Haematochezia	Haemorrhagic colitis	No active bleeding, but there were multiple shallow ulcers with exudate and erythematous lesions on the mucosa involving the entire colon	Crypt architecture was well-preserved and many lymphocytes infiltrated the lamina propria	Dasatinib stopped	Dasatinib restarted at 100 mg/day but symptoms recurred and dasatinib was stopped
Case 2	52 Male	CML	DMR	100 mg/day	1460	Diarrhoea, Bloating	Colitis	Chronic active colitis from the descending colon to the sigmoid, with the rectum not significantly involved; multiple <1 cm sessile polypoidal lesions in the ascending colon and caecum with white exudates on the surface; some loss of vascular marking, colonic erosions and erythema are seen	Mild to moderate chronic active colitis	Dasatinib slowly tapered down to 70 mg then to 50 mg daily	Symptoms resolved
